# Construction, Characterization, and Application of a Nonpathogenic Virus-like Model for SARS-CoV-2 Nucleocapsid Protein by Phage Display

**DOI:** 10.3390/toxins14100683

**Published:** 2022-10-04

**Authors:** Yuting Wu, Bing Liu, Zhiwei Liu, Pengjie Zhang, Xihui Mu, Zhaoyang Tong

**Affiliations:** State Key Laboratory of NBC Protection for Civilian, Beijing 102205, China

**Keywords:** SARS-CoV-2, nucleocapsid protein, phage display, M13 phage, p3 protein

## Abstract

With the outbreak and spread of COVID-19, a deep investigation of SARS-CoV-2 is urgent. Direct usage of this virus for scientific research could provide reliable results and authenticity. However, it is strictly constrained and unrealistic due to its high pathogenicity and infectiousness. Considering its biosafety, different systems and technologies have been employed in immunology and biomedical studies. In this study, phage display technology was used to construct a nonpathogenic model for COVID-19 research. The nucleocapsid protein of SARS-CoV-2 was fused with the M13 phage capsid p3 protein and expressed on the M13 phages. After validation of its successful expression, its potential as the standard for qPCR quantification and affinity with antibodies were confirmed, which may show the possibility of using this nonpathogenic bacteriophage to replace the pathogenic virus in scientific research concerning SARS-CoV-2. In addition, the model was used to develop a system for the classification and identification of different samples using ATR–FTIR, which may provide an idea for the development and evaluation of virus monitoring equipment in the future.

## 1. Introduction

With the outbreak and development of the COVID-19 pandemic since the end of 2019, the world is experiencing an ongoing unprecedented global health crisis. Because of the high pathogenicity and infectiousness of SARS-CoV-2 and the continuous emergence of coronavirus variants, it is more difficult to control the pandemic in a short time, implying that our battle against the COVID-19 pandemic remains long, arduous, and uncertain. Due to the high infectivity, pathogenicity, and instability of the virus, it is difficult to use the virus directly for scientific research. Therefore, multiple technologies have been proposed to synthesize a substitute in order to provide biosafety for scientific research and applications. For example, recombinant adenovirus type-5 (Ad5)- and type-9 (Ad9)-vectored COVID-19, which could express the spike glycoprotein of SARS-CoV-2, were produced and used for immune induction [1,2]. According to Ki-Back Chu et al., by transfecting Sf9 cells, SARS-CoV-2 virus-like particles (VLPs) expressing spike (S) glycoprotein (S full), S1, and S2 were constructed with the expectation of application in vaccine development. The developed VLPs were validated and confirmed by Western blotting and transmission electron microscopy (TEM) [3]. Furthermore, Kim described the platforms of nanoparticle (NP) and VLP technology for developing safer and more effective vaccines, since both NP and VLP vaccines have presented outstanding abilities in immunogenicity and protection through imitating the original characteristics of the SARS-CoV-2 virus [4]. Apart from that, Xing et al. presented their achievements in displaying the receptor-binding domain (RBD) of the S protein from the original SARS-CoV-2 and its three variants on the surface of a saccharomyces cerevisiae, and they claimed its possibility for mass production for SARS-CoV-2 vaccine preparation [5].

Out of the many possible technologies available, phage display is a relatively mature technology that could allow the foreign protein to fuse into the phage capsid protein and display it on the surface [6,7,8]. The exogenous DNA sequences on the phage surface still maintain independent spatial structure and biological activity, which may provide a direct link between phenotype and genotype. Since it was first proposed in the late 1950s by George P. Smith [9], phage display technology has been widely used to exhibit polypeptides, proteins, and panning antibodies. It is popular for its simple operation, high efficiency, and low cost. More specifically, in the research of major infectious diseases such as coronavirus, Flego et al. isolated the nucleocapsid protein of SARS-CoV by applying the single-chain fragment variable (scFv) ETH-2 phage antibody library in 2005, when SARS was widespread. The isolated antibodies and their specificity toward N proteins were confirmed through enzyme-linked immunosorbent assay (ELISA), Western blotting (WB), and immunocytochemistry [10]. Additionally, some reported studies have used phage display in COVID-19 research. Petrenko et al. described a model termed “phage mimicry” to display the viral receptor-binding site on the spike protein by phage display and by employing recombinant filamentous bacteriophages. They used the model to investigate the binding mechanism of the virus to the corresponding receptor in vitro [11]. Further, Yang et al. used phage display technology to research the specific binding of SARS-CoV-2. The SARS-CoV-2-specific peptide they selected showed unique binding ability with the SARS-CoV-2 receptor-binding domain. The binding affinity constant and TEM investigation revealed the possibility of rapid and accurate SARS-CoV-2 modeling [12]. In addition, Somasundaram et al. discussed using a chicken egg-yolk antibodies platform to produce highly specific monoclonal antibodies against SARS-CoV-2 by phage display [13]. Apart from those, some studies have applied phage display technology to express the proteins of SARS-CoV-2. Phage display for the S protein of SARS-CoV-2 was performed by Xiaokang Bai et al. (National Invention Patent of China, Application Number: 202110906836.8). They fused the RBD domain of the S protein to the M13 phage capsid protein and displayed it on its surface. The specific binding ability to the corresponding antibody was verified by enzyme-linked immunosorbent assay (ELISA). Currently, most studies of phage display against SARS-CoV-2 focus on the expression of proteins, investigation of receptor sites, and the development of vaccines and clinical treatments. However, as a highly infectious and pathogenic virus, it is important to detect and identify possible biosecurity threats regarding SARS-CoV-2.

Rapid virus recognition will help ease the pandemic situation and benefit vaccine development. The widely used PCR and ELISA have some advantages towards the detection of the virus [14,15,16,17], but they still have some disadvantages, such as high cost and low efficiency. Currently, research on viruses and spectra, including discrimination and identification of viruses by spectra and algorithms, is commonly investigated. The techniques used in research on the spectral properties of viruses include Raman spectroscopy [18,19,20], infrared spectroscopy [21,22,23,24,25], fluorescence spectroscopy [26], laser-induced breakdown spectroscopy [27], UV/Vis spectroscopy [28], etc. Valerio et al. developed an ultrafast, reagent-free, and non-destructive attenuated total reflection–Fourier transform infrared spectroscopy (ATR–FTIR) method, realizing COVID-19 virus-infected samples being stoichiometrically analyzed [21]. Pizarro et al. combined the FTIR technique with linear discriminant analysis (LDA) to study changes in the plasma metabolic profile of human immunodeficiency virus-infected patients and differentiate it from healthy and subjects infected with different stages of HIV [23]. Santos et al. explored the use of ATR–FTIR technology to identify and quantify dengue virus in blood and serum, utilizing algorithm tools such as principal component analysis (PCA) and LDA, which can distinguish blood and serum samples from different levels of infection [24].

**Figure 1:**
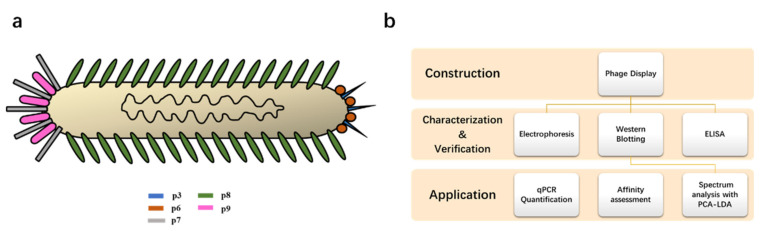
(**a**) Basic structure for the M13 phage. (**b**) Flow diagram of this research from the construction of the model, its characterization, and application.

Considering the current research, we attempted to construct a nonpathogenic model by using the M13 phage to display the N protein of SARS-CoV-2. The M13 phage is a commonly used bacteriophage for phage display. It belongs to a kind of flexible filamentous virus and has host specificity for *E. coli*. The M13 phage has five different capsid proteins, which are p3, p6, p7, p9, and p8. p7, p9, p3, and p6 are distributed on both ends of the phage in pairs, while p8 is wrapped on the surface in a spiral form [29,30] (Figure 1a). The capsid proteins used to construct the display system in the M13 phage are commonly p3 and p8. p3 is distributed at one end of the phage particle, and exogenous polypeptides/proteins can be inserted into the flexible connecting region of the N terminus. The M13 phage has been widely used in establishing antibody-displaying phage libraries by phage display technology, such as scFvs and whole fragment antigen-binding (Fab) antibodies. These fragments could be fused with p3 of the M13 phages, generating a large library for antibody selection [31]. Usually, p3 is used to display proteins of larger molecular weights. As for the N protein containing 419 amino acids, with a molecular weight of about 48 kDa [32], it plays a vital role in promotion of genome packaging, RNA chaperoning, intracellular protein transport, DNA degradation, interference in host translation, and restriction of host immune responses [32,33,34], which is essential in the development of recombinant virus vaccines and immunology research. In our study, the N protein was designed to be expressed at the p3 end of the M13 phage and detected by WB and ELISA. The model was used as the standard for qPCR quantification and its affinity with antibodies was investigated, confirming the possibility of being an alternative choice in some SARS-CoV-2 studies. Furthermore, the produced model was employed in spectrum research, which might provide an idea for the evaluation of monitoring equipment. The research framework is shown in Figure 1b.

## 2. Results

### 2.1. Confirmation of the Constructed Model

**Figure 2:**
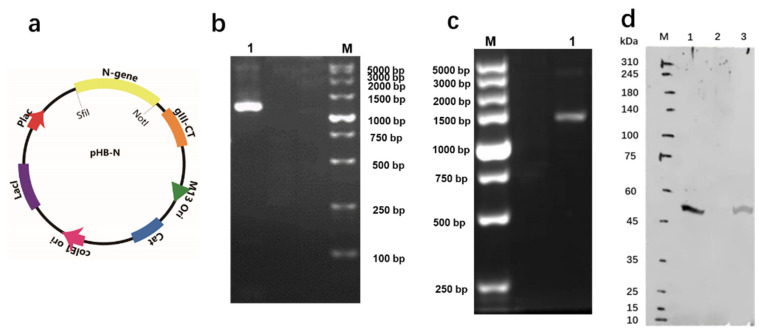
(**a**) Schematic structure for pHB-N. (**b**) PCR amplification results from constructed pHB-N on 1% (*w*/*v*) agarose gel. Lane 1: PCR amplification result for fragment N gene; M: Marker. (**c**) Double digestion from constructed pHB-N on 1% (*w*/*v*) agarose gel. Lane 1: double enzymatic digestion of pHB-N with *Sfi*I and *Not*I; M: Marker. (**d**) Detection of the N protein by Western blotting with anti-SARS-CoV-2 nucleocapsid protein antibody (Rabbit pAb). Lane M: Marker; Lane 1: purchased N protein; Lane 2: original M13 phages; Lane 3: Model-N.

After the preparation and construction of inserted fragments and vectors, pHB-N was produced. (Figure 2a) The constructed pHB-N was identified by PCR amplification (Primer-F: 5′-GGCCCAGCCGGCCATGTCTGATAATGGACCCCAAAATCAG-3′; Primer-R: 5′-ATTTGCGGCCGCTTAGGCCTGAGTTGAGTCAGCAC-3′), double digestion (*Sfi*I/*Not*I), and sequencing (see Supplementary Materials). As Figure 2b,c shows, the bands from PCR amplification and double digestion are consistent with the inserted fragment-prepared N gene (~1271 bp). Therefore, the target gene is proven to be inserted into the vector, and the pHB-N was constructed successfully.

In order to detect the expression of the N protein by phage display, Western blotting was applied, and the results are shown in Figure 2d. The band on Lane 3 of the synthesized phages may indicate an interaction with the anti-SARS-CoV-2 nucleocapsid protein antibody, representing the successful expression of the N protein. Meanwhile, ordinary M13 phages have no sign of any interaction with the antibody (Lane 2). Moreover, compared with the purchased N protein depicted in Lane 1, the same position (~48 kDa) could also prove the reliability of the result.

**Figure 3:**
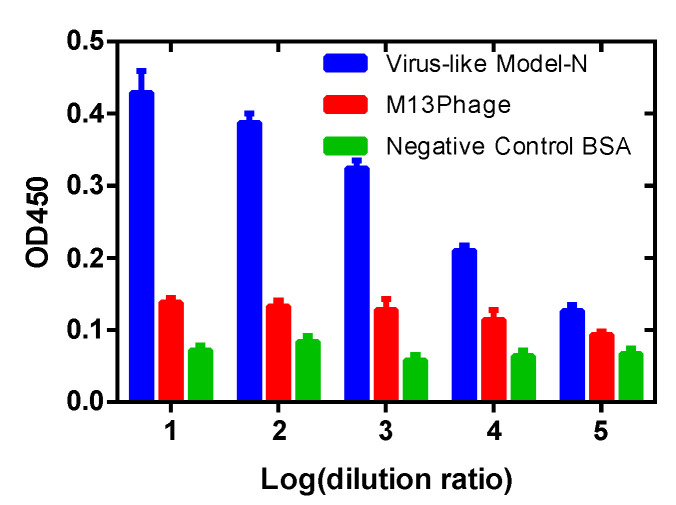
ELISA results for the detection of whether N protein is expressed on the constructed phages or not. BSA and ordinary M13 phages were employed as the references for comparison. Model-N shows a positive reaction with anti-N antibodies, and with an increase in dilution ratio, the absorbance decreases; ordinary M13 phages and BSA are both negative for the combination with anti-N antibodies at all concentrations, showing no reaction with the corresponding antibodies.

Furthermore, sandwich ELISA was employed to determine the expression of the N protein on the synthesized displayed phages (Figure 3). Compared with the negative control BSA (green in Figure 3) and ordinary M13 phages (red in Figure 3), the constructed Model-N phages are positive for the corresponding antibodies. The comparison may indicate the expression of the N protein on the M13 phage by phage display. Therefore, the successful construction and expression of the nonpathogenic model were confirmed.

### 2.2. Characterization of the Constructed Model

#### Titer Determination

The results from the plaque assay are shown in Table 1. The titer is determined as 1.60 × 10^9^ pfu/mL after calculation. Since the titer of the purchased original M13 phage was measured as 8.40 × 10^10^ pfu/mL (NEB product label: 1.0 × 10^11^ pfu/mL), the infectivity of constructed phages decreased by 1–2 orders of magnitude. However, they still maintain the ability to infect bacteria as a virus.

### 2.3. Application for the Constructed Model

#### 2.3.1. Establishment of qPCR Standard Curve

**Figure 4:**
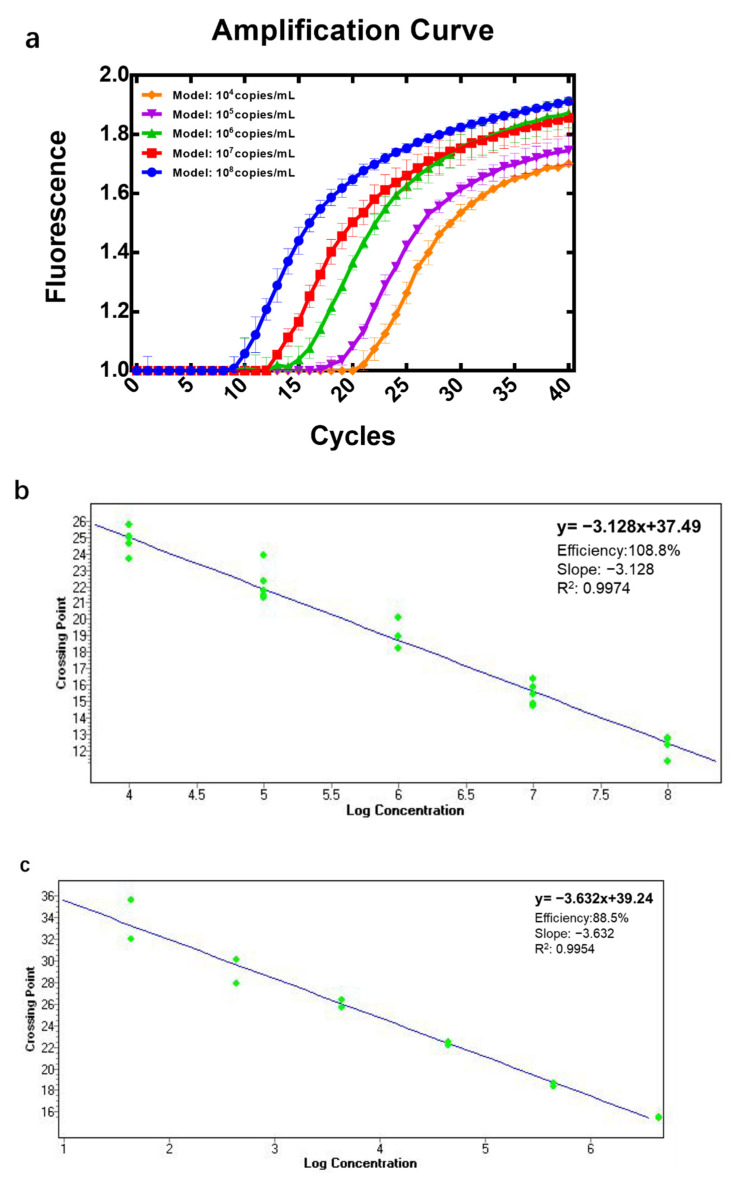
Application of Model-N and establishment of qPCR standard curves. (**a**) Real-time qPCR amplification curve for the constructed Model-N with different copies. The produced Model-N was serially diluted into 5 different concentrations from 10^4^ to 10^8^ copies/mL. All 5 samples presented good amplification and a series of amplification curves were obtained according to the sequence of initial concentration from most to least. The higher the concentration of the sample, the sooner the amplification product reaches the detection value and the fewer cycles they need. (**b**) Standard curve from constructed Model-N. The real-time PCR of serial dilution of Model-N with known concentrations was performed, the standard curve can be made from the linear relationship between the CP (crossing point) value and the logarithm of the concentration of initial templates. Once a sample with unknown concentration can be performed with qPCR and the CP value can also be measured, the initial template amount of the sample can be obtained by substitution into the standard curve. (**c**) Standard curve from purchased standard plasmid. Commercial plasmids were also applied to establish qPCR standard curve, in addition to using the Model-N we made. The regression equation is CP = − 3.632 × log(conc) + 39.24 with R^2^ = 0.9954.

In order to investigate the usage of the nonpathogenic model, qPCR quantification was studied. The constructed recombinant plasmid was quantified and diluted in five gradients. The real-time qPCR reaction was performed with serially diluted plasmid as the template, and amplification was completed (Figure 4a). The standard curve and regression equation were generated by software. The coefficient of standard curve reached 0.9974, indicating a good linear relationship between the template concentration and the CP (Figure 4b). The regression equation is CP = −3.128 × log(conc) + 37.49. Therefore, the initial template concentration can be obtained from the corresponding CP value. R^2^ > 0.99 represents a good linear relationship for both standard curves in the two regression analyses. Compared with Model-N from the purchased standard plasmid (Figure 4c), the standard curve from our constructed model has a more reasonable efficiency (90–110%), revealing the possibility for the constructed model to serve as the quantification standard for qPCR of N genes in COVID-19.

#### 2.3.2. Affinity Analysis

For quantitative detection of intermolecular interactions between Model-N and the anti-SARS-CoV-2 nucleocapsid protein antibody, interaction analysis was conducted using the molecular interaction instrument Fortebio Octet K2. As mentioned in Section 4.4.2, its principle is bio-layer interferometry (BLI) technology, which uses probe-type biosensors to detect samples directly without any fluorescence or isotope labeling. The instrument emits white light to the sensor surface and collects the reflected light. The reflected spectrum of different frequencies is affected by the thickness of the optical film layer of the biosensor and forms interference. Therefore, once the number of molecules bound to the sensor surface increases or decreases, the spectrometer will detect the displacement of the interference spectrum in real time, and this displacement can directly reflect the changes in the thickness and density of the bio-layer on the sensor surface, realizing accurate quantitative determination of the interaction process. Through real-time monitoring of the molecular binding process, the system will determine the association constant and dissociation constant, as well as the initial binding rate, and obtain affinity (KD) and concentration information through fitting calculation analysis.

**Figure 5:**
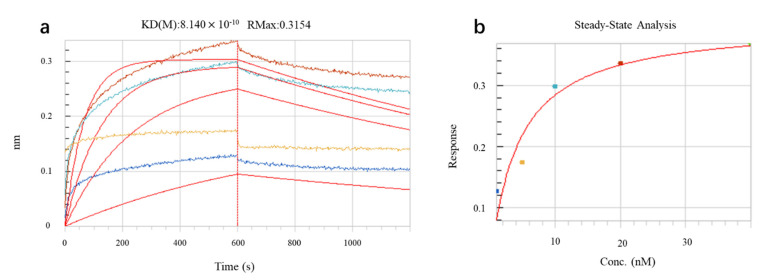
Interaction analysis for Model-N and antibodies. (**a**) Interference shifts over time monitored by sensor. Each colored rough curve represents the shifts in interference spectrum as Model-N and antibodies associate or disassociate. The four red smooth curves are fitting results of the original outcomes. (**b**) Response change over concentration of Model-N. The response shows an upward trend as the concentration increases.

The reaction between Model-N and corresponding antibodies was monitored and recorded, as Figure 5a shows. Four samples with different concentrations were employed in this experiment and each colored line in Figure 5a represents one sample. As the figure indicates, the interference spectrum (*Y*-axis) shifts over time (*X*-axis). For each line, the rising section describes the association process between Model-N and antibodies, while the descending section can explain the disassociation. In addition, the red smooth curves are the fitting results for the original experimental outcomes by software. Figure 5b illustrates the relationship between concentrations of Model-N and the response (monitored by the sensor). The response intensities enhance as the concentrations increase, exhibiting an upward trend in curve fitting. After fitting analysis and repetition, the affinity constant is measured as 1.229 × 10^9^ M^−1^, which may indicate a strong combination between Model-N and anti-N-protein antibodies.

Through the above analyses, it could be proven that the N protein of SARS-CoV-2 has been expressed on the displayed phages of Model-N successfully. It has a relatively strong combination with anti-N-protein antibodies. Therefore, the constructed phage Model-N might possibly be used as a nonpathogenic model for immunology and biomedical research of SARS-CoV-2. On the one hand, the biological properties of the virus species are preserved and maintained; on the other hand, the properties of the N gene and N protein were both validated and confirmed on molecular and protein levels; the constructed model can probably be used as a biosafe substitute of SARS-CoV-2 for some research.

#### 2.3.3. ATR–FTIR Combined PCA-LDA Algorithm to Classify and Identify Substance Bio-Threats Such as Model-N

This section attempts to provide a technical system for virus monitoring, classification, and identification based on the infrared spectral characteristics of Model-N and other relative materials. Specifically, since Model-N has been verified and confirmed at molecular and protein levels, we attempt to apply it in a spectrum study and utilize a PCA-LDA algorithm to provide a possible verification and performance evaluation idea for the development of real-time virus monitoring and early-warning equipment.

**Figure 6:**
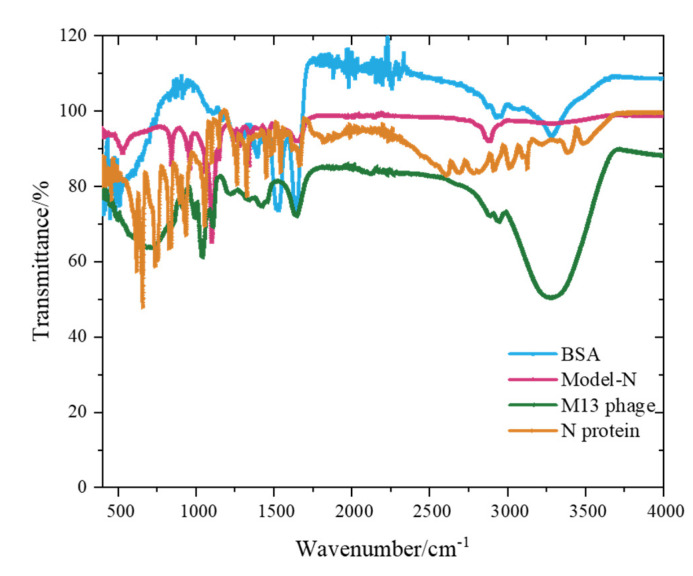
ATR–FTIR spectrum of four selected materials. The numbers of scans for the sample and background were both 64, and the wavenumber range was 400–4000 cm^−1^.

Model-N, the M13 phage, the commercial SARS-CoV-2 N protein, and the BSA protein were selected for this study. ATR–FTIR was employed and data were collected using a thermo-fluorescence spectrometer (Figure 6).

After data collection, principal component analysis (PCA) was performed for dimensionality reduction (see Table 2). The cumulative percentage of the first three principal components reached 84.220%, with less than 16.000% of information lost. A cumulative percentage over 80% represents a good dimensionality reduction. Therefore, the first three principal components were used to describe raw data.

**Figure 7:**
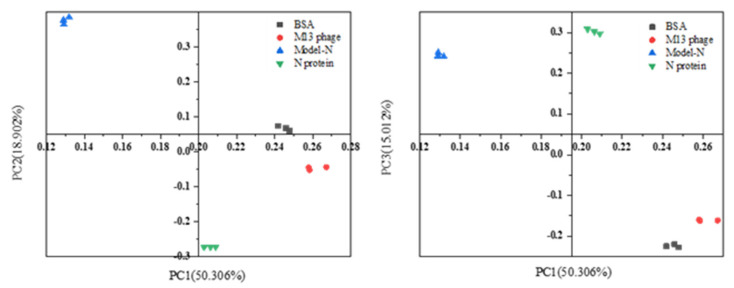
Scatter plots of components’ score for the four samples from the ATR-FTIR spectrum. Coordinate systems are composed of PC1-PC2 and PC1-PC3. After PCA analysis, the four samples, Model-N, M13 phage, commercial SARS-CoV-2 N protein, and BSA protein are separated with distinct differences, which may imply a significant cluster variation.

**Figure 8:**
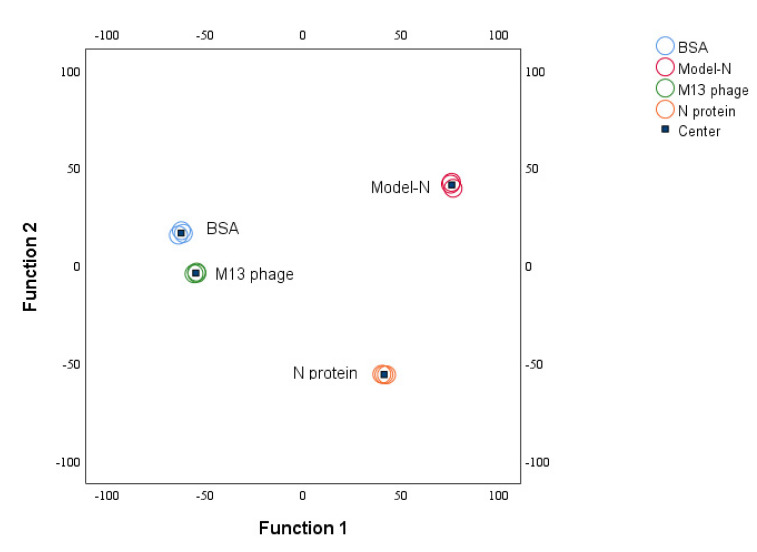
Group centroid plot from LDA analysis. All four samples were separated and classified. Training samples for every group were concentrated well.

According to the scatter plots, it can be seen that in the coordinate systems composed of PC1–PC2 and PC1–PC3 (see Figure 7), the four substances were concentrated in different regions with distinguished differentiation. The obvious category difference implied the possibility that the four samples could be used as training samples for discriminant analysis. Therefore, the first three principal components were added as training samples and applied with linear discriminant analysis (LDA). The discriminant functions were obtained (see Supplementary Materials File S1), and a centroid plot was created (Figure 8). The distribution of training samples for every group was concentrated, and different groups were well-separated, indicating that the classification, discrimination, and identification of the four substances could be achieved after PCA-LDA analysis.

In summary, the prepared Model-N was applied in this section. Combined with ATR–FTIR and a PCA-LDA algorithm, the identification and classification of possible bio-threat substances were realized. This section is not only concerned with the application of the constructed model, but also provides an idea for the development and evaluation of virus monitoring equipment in the future. When new virus monitoring equipment needs to be simulated or tested, the prepared nonpathogenic virus-like model can be used as a reference/sample to evaluate the performance of the equipment without any contamination or hazards.

## 3. Conclusions

In this study, we used phage display technology to fuse the nucleocapsid protein of SARS-CoV-2 to the M13 phage’s p3 protein. From the molecular study level, we verified the successful insertion of the target fragment; from the protein study level, we confirmed that the synthesized phages could express the N protein through Western blotting and ELISA. In addition, we described its applications differently. Firstly, we investigated the qPCR quantification and affinity interaction using the constructed model to imply the possibility of being a substitute in COVID-19 research. The standard curve was established successfully, and the affinity constant reached 1.229 × 10^9^M^−1^, proving a strong interaction with anti-N-protein antibodies. We confirmed the possibility of using this nonpathogenic model instead of the pathogenic virus in COVID-19 studies. Furthermore, as a type of virus, the infectivity of the synthesized phages was maintained, and the biological properties of the virus species were preserved. Compared with the commercial N protein, the model is a kind of virus which may possess more biological properties akin to SARS-CoV-2. Therefore, the model we constructed might be a useful nonpathogenic substitute for SARS-CoV-2 for some research.

Furthermore, we employed the model in classification and identification of potential “bio-threats” with ATR–FTIR and algorithms. The results showed that the four relative samples, including the bio-threat Model-N, were classified well after PCA-LDA analysis and might be useful in developing and evaluating virus monitoring equipment in the future. Additionally, our research provides a new idea to study this highly pathogenic and infectious virus in a safe environment and confirms its feasibility.

## 4. Materials and Methods

### 4.1. Materials and Instruments

#### 4.1.1. Materials

The constructed phagemid vector pHB was donated by the Academy of Military Medical Sciences, and contained the chloramphenicol resistance gene, the plac promoter, the gIII gene of M13, and the *Sfi*I/*Not*I site for cloning the target gene [14]. *Sfi*I, *Not*I enzymes, and M13KO7 helper phages were purchased from New England Biolabs (Ipswich, MA, USA). *E. coli* TG1 and nuclease-free water were bought from Biomed (Beijing, China). PrimeSTAR HS (Premix) for PCR amplification, T4 DNA ligase, and a DL5000 DNA Marker were purchased from TaKaRa (Kusatsu, Shiga, Japan). Agarose, kanamycin, chloramphenicol, BSA, PBS buffer, TBST buffer, TAE buffer, ELISA coating buffer, stop buffer, TMB single-component substrate solution, and SARS-CoV-2 N protein were bought from Solarbio (Beijing, China). A PureYield™ Plasmid Miniprep System and a Wizard^®^ SV Gel and PCR Clean-Up System were bought from Promega (Madison, WI, USA). Poly (ethylene glycol) (BioUltra 8000, CAS: # 25322-68-3) was bought from Sigma-Aldrich (St. Louis, MO, USA). Rabbit anti-SARS-CoV-2 N-protein polyclonal antibody and IRDye^®^ 800CW goat anti-rabbit IgG (H + L) were bought from Bioss. Rabbit anti-M13 pAb-HRP was bought from Beijing Antaizhiyuan Technology Co., Ltd. (Beijing, China). Skim milk powder was bought from Anchor. Syringe filter units (0.45 µm) were bought from Merck Millipore (Darmstadt, Germany). A nitrobiocellulose filter membrane was bought from Bio-Rad (Hercules, CA, USA). All broths and culture media were prepared in our lab. N gene standard plasmid (for qPCR) was bought from Shanghai Institute of Measurement and Testing Technology (Shanghai, China).

#### 4.1.2. Instruments

A PCR amplifier was bought from Thermo Fisher (Waltham, MA, USA). Electrophoresis was implemented by a Bio-Rad apparatus (Hercules, CA, USA). An automatic microplate reader (SPARK) was bought from TECAN (Männedorf, Switzerland). An Odyssey^®^ Imaging system was from LI-COR (Lincoln, NE, USA). A molecular interaction analyzer (ForteBio Octet K2) was bought from SARTORIUS (Gottingen, Germany). ATR–FTIR was bought from Thermo (Waltham, MA, USA).

### 4.2. Model-N Construction

#### 4.2.1. Preparation for the Target Gene: PCR Amplification and Double Endonuclease Digestion

The gene corresponding to the N protein of SARS-CoV-2 was investigated at the National Center for Biotechnology Information (NCBI website/Gene ID: 43740575). The original sequence was redesigned and synthesized, including the addition of *Sfi*I/*Not*I restriction digest sites at both ends. Additionally, the corresponding primers were designed and synthesized according to the redesigned target genes (F: 5′-TCTGATAATGGACCCCAAAATCAG-3′; R: 5′-ATTTGCGGCCGCTTAGGCCTGAGTTGAGTCAGCAC-3′). Then, PCR was employed in a 50 μL reaction system, which contained 25 μL PrimeSTAR HS (Premix), 50 pmol/μL primers for both ends, 1 μL template plasmid, and 23 μL nuclease-free water. The PCR amplification reaction program was set at 95 °C/10 min for initial denaturation, then followed by 30 cycles at 94 °C/1 min for denaturation, 56 °C/1 min for annealing, 72 °C/90 s for extension, and another 72 °C/10 min for a final extension. After purification, the obtained genes were digested with *Sfi*I and *Not*I, sequentially. Because of the different reaction temperatures and buffer environments for the two enzymes, a step-by-step procedure was required. The compositions are displayed in Table 3.

The produced fragments from double enzyme digestion were purified and named prepared-N-gene.

#### 4.2.2. Preparation for the Vector: Double Endonuclease Digestion

Similarly, the phagemid vector pHB-1HSCFV was digested by the same enzymes *Sfi*I and *Not*I, employing conditions similar to mentioned above. Following purification and recovery after digestion, the vector was named prepared-pHB and prepared for construction.

#### 4.2.3. Construction for the Recombinant Vector

After the preparations of inserted fragments and vectors, the recombinant vectors were constructed following the conditions in Table 4, and the produced results were named pHB-N.

#### 4.2.4. Cultivation and Phage Display

The recombinant pHB-N was transformed into the competent *E. coli* TG1 cells. Then, 100 μL TG1 was inoculated in 5 mL of 2xTY broth with chloramphenicol (34 mg/mL) and cultivated overnight at 37 °C with a shake rate of 250 rpm. The following day, the cultivated mixture was inoculated into 40 mL of 2xTY broth at a ratio of 1:100 and cultivated at 37 °C to its logarithmic growth phase while shaking. Then, the M13KO7 helper phages were added with a titer of1 × 10^9^–4 × 10^9^ pfu/mL. The mixture was cultivated at 37 °C at 150 rpm for 2 h. After centrifuging at 4000× *g* for 10 min, the precipitates were resuspended in 200 mL of 2xTY broth containing kanamycin (25 mg/mL). The culture mixture was cultivated overnight at 30 °C at 250 rpm. Then, after centrifugation (4000× *g*/4 °C/10 min), 20% polyethylene glycol 8000/2.5 M NaCl was added to the supernatant and shaken violently. Another centrifugation (9000× *g*/4 °C/20 min) was employed after a one-hour incubation on ice, and the pellet was resuspended with 2–3 mL of PBS and filtered through a 0.45 μm filter membrane. The constructed products were stored at 4 °C.

### 4.3. Model-N Characterization and Verification

#### 4.3.1. Insertion of the Gene Fragments

The constructed pHB-N was identified by PCR amplification, double digestion (*Sfi*I/*Not*I), and sequencing to verify the insertion of the target gene. The electrophoresis results were used to determine whether the target genes were inserted or not.

#### 4.3.2. Western Blotting

Western blotting was employed to detect whether the N protein of SARS-CoV-2 was expressed on the synthesized phage display. Western blotting, also known as immunoblotting, is a method of using blotting technology to transfer proteins to membranes and then detect particular proteins in complex samples according to the specific binding of antigens and antibodies. Firstly, the mixed protein samples were separated by polyacrylamide gel electrophoresis (PAGE), and then blotted onto a solid-phase medium (such as a nitrocellulose (NC) membrane). Then, the protein or polypeptide on the solid-phase carrier was used as an antigen, which may have specific binds with the primary antibody. After that, the mixture was incubated with the enzyme or isotope-labeled secondary antibody. The target protein could be observed by substrate color development or autoradiography. Regular steps of Western blotting were applied. First of all, electrophoresis was accomplished with the Bio-Rad apparatus. Then, membrane transfer with a 0.45 μm NC filter membrane was achieved, followed by blocking with 5% skim milk powder/BSA for 1 h at room temperature. Then, rabbit anti-SARS-CoV-2 N-protein polyclonal antibody was used for primary antibody incubation and IRDye^®^ 800CW goat anti-Rabbit IgG (H + L) was employed for secondary antibody incubation. Followed by washing 4 times with TBST, color development was performed and observed by the Odyssey^®^ Imaging system.

#### 4.3.3. ELISA

Sandwich ELISA was used to detect if there was any sign of the expression of N proteins of displayed phages. A 96-well plate was coated with rabbit anti-SARS-CoV-2 N-protein polyclonal antibody. After washing and blocking, the displayed phages with different diluted ratios were added for binding. Then, the rabbit anti-M13 pAb-HRP was added and combined to form a sandwich structure. After adding TMB substrate, the OD values at 450 nm were obtained by the automatic microplate reader.

#### 4.3.4. Titer Determination

The gold standard method for titer determination, plaque assay, was employed to determine the titer of the synthesized phages. The displayed phages with different dilution ratios were used to infect *E. coli* TG1, and the mixture was placed on LB solid culture media (with kanamycin). The cultures were cultivated overnight at 37 °C, and all formed plaques were counted the next day.

### 4.4. Model-N Application

#### 4.4.1. qPCR Quantification

Real-time qPCR standard curves were established for the purchased standard plasmid containing the N gene and the recombinant plasmid pHB-N of the construction model (see Table 5). The possibility for the constructed model as the standard in qPCR quantification was attempted and confirmed. (Primer-F: GGGGAACTTCTCCTGCTAGAAT; Primer-R: CAGACATTTTGCTCTCAAGCTG; Probe: 5′-VIC-TTGCTGCTGCTTGACAGATT-BHQ1-3′).

#### 4.4.2. Affinity Assessment

The molecular interaction instrument Fortebio Octet K2 was used to determine the affinity of the expressed N protein on the synthesized phage and its antibody [35,36]. The instrument was based on bio-layer interferometry (BLI) technology, which is a technique that reflects the surface response of a sensor by detecting the displacement change in the interference spectrum. Its working principle is that when a beam of visible light is emitted from the spectrometer, two beams of reflection spectra will be formed at the interfaces of the optical film layer, and one beam of interference spectrum will be obtained. Any changes in the thickness and density of the film formed by molecular binding or dissociation can be reflected by the displacement value of the interference spectrum, and a real-time response-monitoring map can be made through this displacement value [37,38]. In this experiment, the first step was to add curing buffer PBST to the pre-wet plate, and the sensor was placed on it; secondly, samples such as Model-N with different dilution ratios, the ligand (anti-SARS-CoV-2 nucleocapsid protein antibody (rabbit pAb)), and the regenerated solution (glycine) were all prepared on the sample plate; thirdly, the program was set to ensure that each sample could undergo the steps of baseline, loading, association with antibodies, dissociation, regeneration, etc. All processes could be monitored, and important data such as association constant (ka) and dissociation constant (kd) could be obtained and analyzed. Then, the affinity constant, which could measure the interaction quantitatively between Model-N and corresponding antibodies, was calculated.

#### 4.4.3. Classification and Identification of Model-N by ATR–FTIR

Model-N, as a synthetic phage, was designed to display the N protein of SARS-CoV-2. It could be used as a potential bio-threat when developing or evaluating the performance of real-time virus monitoring and early-warning equipment. In this section, Model-N, the M13 phage, the commercial SARS-CoV-2 N protein, and the BSA protein were selected to employ ATR–FTIR (using a thermo-fluorescence spectrometer). The data were collected and processed with the PCA-LDA algorithm to realize the classification and recognition of the four samples. All data were analyzed by IBM SPSS Statistics 26.0.

## Figures and Tables

**Table 1:** The titer calculation for Model-N.

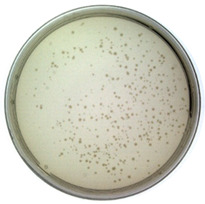	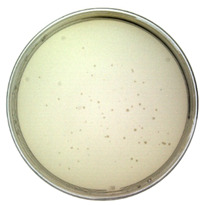	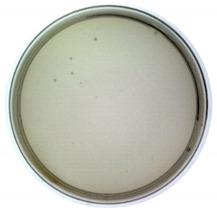
Dilution ratio: 10^5^	Dilution ratio: 10^6^	Dilution ratio: 10^7^
The number of plaques: uncountable	The number of plaques: 70	The number of plaques: 9
Titer: 1.60 × 10^9^ pfu/mL		

**Table 2:** Analysis and explanation table by PCA.

Principal Component	Initial Eigenvalues	
	Percentage of Variance/%	Cumulative Percentage/%
1	50.306	50.306
2	18.902	69.208
3	15.012	84.220
4	10.114	94.334
5	2.372	96.706
6	1.147	97.853

Note: data of principal components are omitted after the 6th.

**Table 3:** Double digestion for the inserted fragments. (**a**) *Sfi*I conditions; (**b**) *Not*I conditions.

(**a**) *SfiI Digestion System*	
Target gene (after PCR)	20 μL
10× buffer1	8 μL
*Sfi*I enzyme	4 μL
Nuclease-free water	43 μL
Total volume	75 μL
Condition: Incubate at 50 °C for 4 h	
** (**b**) *NotI digestion system***	
System after *Sfi*I digestion	75 μL
10× buffer2	10 μL
*Not*I enzyme	4 μL
Nuclease-free water	11 μL
Total volume	100 μL
Condition: Incubate at 37 °C for 4 h	

**Table 4:** Compositions for the construction system.

Construction System for pHB-N	
Prepared-N-gene	10 μL
Prepared-pHB	3 μL
10× buffer	5 μL
T4 DNA Ligase	2 μL
Total volume	20 μL
Condition: Incubate at 16 °C overnight	

**Table 5:** Compositions for qPCR amplification and quantification.

qPCR Reaction	
Plasmid	2 μL
Buffer	10 μL
Primer-F	0.32 μL
Primer-R	0.32 μL
Taqman (VIC)	2 μL
Enzyme 2	0.4 μL
Enzyme 3	0.4 μL
Nuclease-free water	4.56 μL
Total volume	20 μL

## Data Availability

Not applicable.

## Supplementary Materials

The following supporting information can be downloaded at: https://www.mdpi.com/article/10.3390/toxins14100683/s1, File S1: Construction, characterization and application of a nonpathogenic virus-like model for SARS-CoV-2 nucleocapsid protein by phage display Supplementary Materials.
