# Supplementary material for: Construction, Characterization, and Application of a Nonpathogenic Virus-like Model for SARS-CoV-2 Nucleocapsid Protein by Phage Display
Source: Toxins (Basel). 2022 Oct 4;14(10):683. doi: 10.3390/toxins14100683 (PMC9607219; doi:10.3390/toxins14100683)
Supplement: Supplementary file 1 [file toxins-14-00683-s001.zip › toxins-1906766-supplementary.pdf]

toxins

MDPI

Supplementary Materials

Construction, Characterization and Application of a Nonpathogenic Virus-like Model for SARS-CoV-2 Nucleocapsid Protein by Phage Display

Yuting Wu, Bing Liu, Zhiwei Liu, Pengjie Zhang, Xihui Mu and Zhaoyang Tong *

1.Sequencing result for pHB-N insertion fragment

GGCCCAGCCGGCCATGTCTGATAATGGACCCCAAAATCAGCGAAATGCACCCCGCATTACGTTTGGTGGACC
CTCAGATTCAACTGGCAGTAACCAGAATGGAGAACGCAGTGGGGCGCGATCAAAACAACGTCGGCCCCAAG
GTTTACCCAATAATACTGCGTCTTGGTTCACCGCTCTCACTCAACATGGCAAGGAAGACCTTAAATTCCCTCG
AGGACAAGGCGTTCCAATTAACACCAATAGCAGTCCAGATGACCAAATTGGCTACTACCGAAGAGCTACCA
GACGAATTCGTGGTGGTGACGGTAAAATGAAAGATCTCAGTCCAAGATGGTATTTCTACTACCTAGGAACTG
GGCCAGAAGCTGGACTTCCCTATGGTGCTAACAAAGACGGCATCATATGGGTTGCAACTGAGGGAGCCTTGA
ATACACCAAAAGATCACATTGGCACCCGCAATCCTGCTAACAATGCTGCAATCGTGCTACAACTTCCTCAAG
GAACAACATTGCCAAAAGGCTTCTACGCAGAAGGGAGCAGAGGCGGCAGTCAAGCCTCTTCTCGTTCCTCAT
CACGTAGTCGCAACAGTTCAAGAAATTCAACTCCAGGCAGCAGTAGGGGAACTTCTCCTGCTAGAATGGCTG
GCAATGGCGGTGATGCTGCTCTTGCTTTGCTGCTGCTTGACAGATTGAACCAGCTTGAGAGCAAAATGTCTGG
TAAAGGCCAACAACAACAAGGCCAAACTGTCACTAAGAAATCTGCTGCTGAGGCTTCTAAGAAGCCTCGGC
AAAAACGTACTGCCACTAAAGCATACAATGTAACACAAGCTTTCGGCAGACGTGGTCCAGAACAAACCCAA
GGAAATTTTGGGGACCAGGAACTAATCAGACAAGGAACTGATTACAAACATTGGCCGCAAATTGCACAATTT
GCCCCCAGCGCTTCAGCGTTCTTCGGAATGTCGCGCATTGGCATGGAAGTCACACCTTCGGGAACGTGGTTGA
CCTACACAGGTGCCATCAAATTGGATGACAAAGATCCAAATTTCAAAGATCAAGTCATTTTGCTGAATAAGC
ATATTGACGCATACAAAACATTCCCACCAACAGAGCCTAAAAAGGACAAAAAGAAGAAGGCTGATGAAAC
TCAAGCCTTACCGCAGAGACAGAAGAAACAGCAAACTGTGACTCTTCTTCCTGCTGCAGATTTGGATGATTTC
TCCAAACAATTGCAACAATCCATGAGCAGTGCTGACTCAACTCAGGCCTAAGCGGCCGC

2. Functions by linear discriminant analysis

$$f_1 = 63.373 \times PC_1 + 83.666 \times PC_2 + 255.854 \times PC_3 - 26.306$$

$$f_2 = -97.240 \times PC_1 + 132.988 \times PC_2 - 58.845 \times PC_3 + 18.757$$

$$f_3 = 296.542 \times PC_1 + 40.275 \times PC_2 + 50.189 \times PC_3 - 65.731$$
